# Calculating semantic relatedness for biomedical use in a knowledge-poor environment

**DOI:** 10.1186/1471-2105-15-S14-S2

**Published:** 2014-11-27

**Authors:** Maciej Rybinski, José Francisco Aldana-Montes

**Affiliations:** 1 Departamento LCC, University of Malaga, Campus Teatinos, 29010 Malaga, Spain

**Keywords:** Bioinformatics, Semantic relatedness, Semantic similarity, Distributional linguistics, Knowledge extraction

## Abstract

**Background:**

Computing semantic relatedness between textual labels representing biological and medical concepts is a crucial task in many automated knowledge extraction and processing applications relevant to the biomedical domain, specifically due to the huge amount of new findings being published each year. Most methods benefit from making use of highly specific resources, thus reducing their usability in many real world scenarios that differ from the original assumptions. In this paper we present a simple resource-efficient method for calculating semantic relatedness in a knowledge-poor environment. The method obtains results comparable to state-of-the-art methods, while being more generic and flexible. The solution being presented here was designed to use only a relatively generic and small document corpus and its statistics, without referring to a previously defined knowledge base, thus it does not assume a 'closed' problem.

**Results:**

We propose a method in which computation for two input texts is based on the idea of comparing the vocabulary associated with the best-fit documents related to those texts. As keyterm extraction is a costly process, it is done in a preprocessing step on a 'per-document' basis in order to limit the on-line processing. The actual computations are executed in a compact vector space, limited by the most informative extraction results. The method has been evaluated on five direct benchmarks by calculating correlation coefficients w.r.t. average human answers. It also has been used on Gene - Disease and Disease- Disease data pairs to highlight its potential use as a data analysis tool. Apart from comparisons with reported results, some interesting features of the method have been studied, i.e. the relationship between result quality, efficiency and applicable trimming threshold for size reduction. Experimental evaluation shows that the presented method obtains results that are comparable with current state of the art methods, even surpassing them on a majority of the benchmarks. Additionally, a possible usage scenario for the method is showcased with a real-world data experiment.

**Conclusions:**

Our method improves flexibility of the existing methods without a notable loss of quality. It is a legitimate alternative to the costly construction of specialized knowledge-rich resources.

## Background

Given the massive amount of new research that has been published in the life sciences in recent years, the scientific community needs solutions that lead to the creation of self-readable document repositories, which could automatically classify and provide structural representation of the knowledge expressed 'implicitly' (from a machine-based perspective) in the scientific articles. Achieving this goal would be an important step, that eventually could lead to improving current information access and retrieval methods, which are mostly based on keyword queries. The in-adequacy of currently available tools and approaches has been mentioned in the domain literature, e. g. [1]. Establishing semantic relatedness between concepts or their textual representation is one of the key enabling components in automated knowledge extraction from texts, as many text processing applications need a numerical equivalent of how the concepts fit together. According to [2], successfull applications of approximations of semantic relatedness include general domain tasks such as: word sense disambiguation [3], text summarization [4] and information retrieval [5]. According to [6], applications of semantic similarity (which is a more specific concept) and relatedness measures in life sciences include direct data analysis (discovery of protein - pathway interactions [7], discovering similar diseases [8]), semantic search [9], redundancy detection in clinical records [10], sense disambiguation [11]. Applications of semantic similarity to compare gene products have been reviewed in [12].

Most existing state-of-the-art measures use some kind of pre-existing knowledge base in order to produce a numerical approximation of semantic relatedness between two concepts or their lexicalizations, as reflected in a relatively recent survey presented in [13]. This trend has also been reflected in a recent review presented in [14]. It can be argued that the notion of semantic relatedness is connected with such resources due to its concept-based nature [15]. However, as pointed out in [13,16,17], corpus-based methods have been used with some success as approximations of semantic relatedness. To the best of our knowledge, all the state-of-the-art measures that are applied in life sciences make use of highly specialised knowledge-rich resources, which makes them barely suitable for scenarios in which the knowledge base does not exist, or is extremely large or subject to frequent changes. Furthermore, their methodologies are not easy to repeat for even a slight change of settings, as reflecting each change in the knowledge resource would require substantial effort. Our aim is to propose a method that is as general and flexible as possible, with respect to a chosen corpus, given that finding a document collection for a domain is normally a much easier task than creating a comprehensive knowledge base for this domain. As it is reasonable to assume that composing a document collection is relatively easy as long as the collection size remains small to moderate, the method should work with a corpus significantly smaller than the web-scale.

In this paper we present a method, which can be used as an approximation of semantic relatedness for life sciences in knowledge-poor settings. It is purely corpusbased, therefore it does not need any additional artifacts, apart from a domain oriented document collection. Its distributional nature means that the costly pre-processing stage of the method can be executed offline, regardless of the predefined set of entities of interest, on a 'per-document' basis. This means that the setup of the method does not depend on the entities of interest, nor on their quantity, hence the entities can be unknown *a priori*. An atomic goal of the method is to pro-vide a numerical approximation of semantic relatedness for a pair of input queries (represented by terms or their collocations). Our method is based on an idea that contexts of related lexical representations of concepts will be similar ("You shall know a word by the company it keeps", J. R. Firth, 1957), i.e. scientific articles on related concepts should contain similar vocabulary. For each input text we recover a set of best fit documents from the corpus and compose a term vector related to the given input. The term vector is aggregated through the extraction of rep-resentative terms from the best fit documents. The comparison of vectors results in a numerical approximation of semantic similarity between the respective textual representations. This idea is similar to those presented in [18] (general domain) and in [19], only here the vectors are obtained for each document in the preprocessing and then aggregated at runtime for each lexicalization in the function of the most suitable documents. By doing this, our method is not dependent on a pre-existing knowledge base nor on the quantity of the entities of interest, thus making it more adaptable to different use cases, which may not have suitable dictionary/ontological resources. In a sense, we postulate a novel approach for the rough approximation of contexts for relatedness approximation, while existing methods focus more on the preprocessing that enables context extraction for a closed set of entities.

An important feature of the design is that the method can work with very large (possibly too large for 'per instance' processing), flat (with no relationships between the data objects) and dynamic (the evolution of the set does not imply the necessity of re-calibration) sets of entities, possibly unknown until runtime. This characteristic is especially important, given that modern application designs often depend on distributed data provided by third parties. Also, many commonly used databases present only a specific perspective on the possible relationships between their entities, which does not necessarily correspond to the perspective of the intended usage. Moreover, the relationships defined within the databases rarely provide enough structure for approximating relatedness of objects. The method presented here is unaffected by these limitations, and can be used in a dynamic environment for applications such as relatedness based query expansion, where a query for a specific entity is expanded to encompass the objects that are closely related to the entity of interest.

In the following section we present both the method itself, together with its key components, as well as the experimental settings used for its evaluation. In the third section the results of the experiments are presented and discussed. We additionally provide a comparative analysis of two diferrent approaches to document preprocessing. Moreover, we compare the results achieved for two versions of a document corpus, which covers a wide range of Life Sciences domains and subdomains, while being manageable in terms of the corpus size. Remarks on the usability of the method are also presented together with discussion points on corpus driven methods in general. In the last section we summarize the work presented here.

## Methods

### Overview

**Figure 1:**
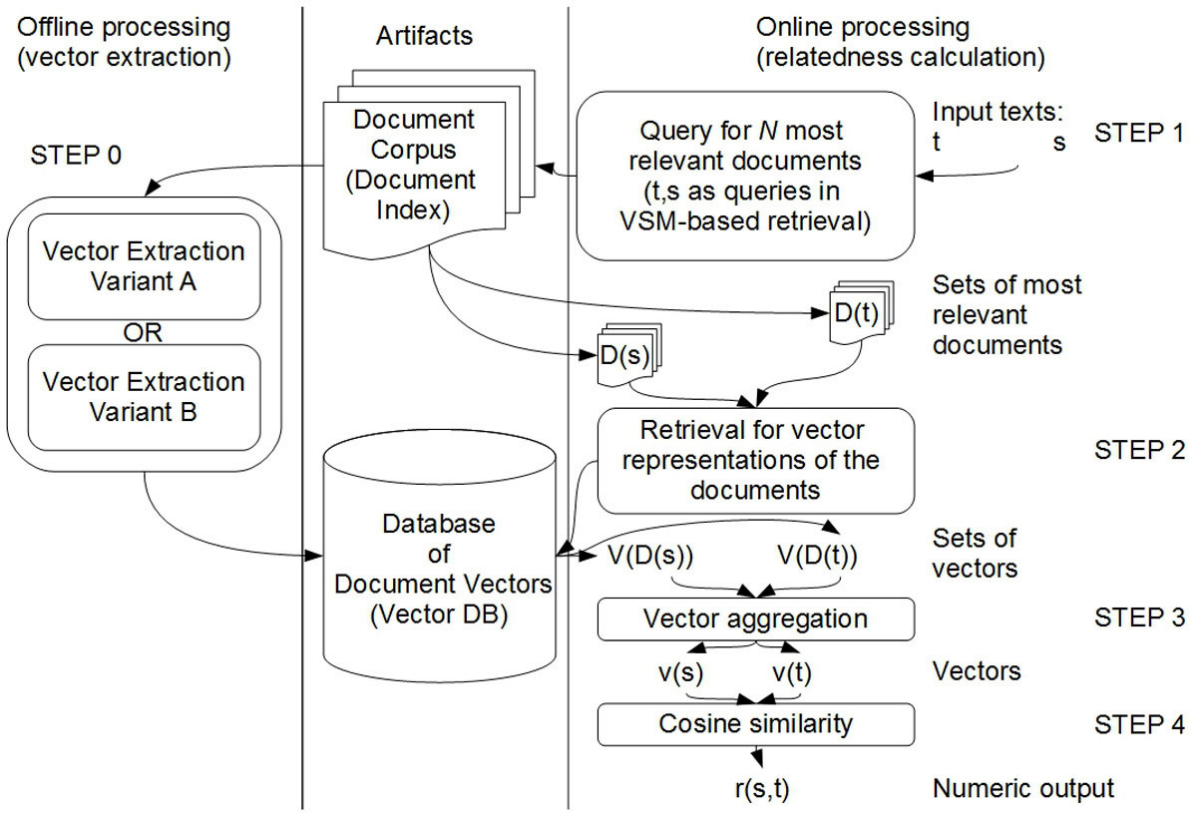
**Overview**. Overview of the method's components and its processing flow; *s *and *t *denote input texts, *D*(*k*) denotes a set of the most relevant documents retrieved for an input *k, V *(*D*(*k*)) denotes a set of the most relevant document vectors for documents retrieved for an input *k, v*(*k*) is a vector aggregated to represent the contexts of input *k *in the relatedness calculation and *r*(*k*_1_*, k*_2_) denotes the relatedness between a pair of input values *k*_1 _and *k*_2_.

As indicated in the previous section, our method uses a document collection of a fairly general nature and moderately large size, with respect to the relative scope and size of the domain of interest. The size and scope of the corpus is discussed in more detail in the Data section of this paper. As the method is expected to deal with free input or very large databases, in cases where per-instance preprocessing would have been too costly, the corpus is the only data used in the preprocessing stage. It was implemented as an Apache Lucene [20] index. In the preprocessing stage each document included in the corpus is analyzed individually so as to extract its most important terms and/or phrases (collocations of terms). The results of this analysis are stored as sparse vectors in a separate database, which is described in detail further on in this section. The database implementations can be based on any persistent key-value map. These two elements, the document corpus and the database of document vectors, are used at runtime to process requests for a given input, i.e. a pair of text labels. A general overview of the components and processing flow (both offline and online) is given in Figure 1.

As already mentioned, the method requires two key components for its online processing, namely the Document Corpus and the Database of Document Vectors. Those artifacts are also referred to as the document index (as the processing requires the corpus to be queryable) and the vector database (vector DB). The runtime input consists of two texts, for which, the relatedness score is to be calculated. First, we establish a set of the most relevant documents for each of the input texts. Second, we calculate a vector representation of the key-phrases associated with the respective document sets. Once the vectors have been extracted, the relatedness score is calculated and returned, thus terminating the procedure. The processing performed for the relatedness calculation for each input pair of texts can be presented in a more structured way with the following breakdown:

**STEP 0 **Step 0 is performed offline and globally, so it is also referred to as a preprocessing step; it takes the Document Corpus as input in order to create its vector-based representation - the vector database.

**STEP 1 **Step 1 is the first online processing step; its goal is to return a set of *N *most adequate documents from the corpus for each of the user-provided inputs; for this purpose the typical information retrieval model is used, i.e. inputs are treated as queries.

**STEP 2 **During Step 2 the sets of the most relevant documents retrieved in the previous step are substituted by sets of vector-based representation of those documents; as obtaining the vectors on the fly would be too costly, they are retrieved from the vector DB created during the preprocessing step.

**STEP 3 **In Step 3 the output from Step 2, i.e. two sets of vectors, is converted into a pair of vectors in an aggregation process; each of these vectors corresponds to one of the inputs.

**STEP 4 **In Step 4 the vectors aggregated in Step 3 are used to calculate the relatedness score between the input texts, which concludes the online processing for a pair of inputs.

The specific elements of the method outlined here are discussed later in this section.

### Preprocessing and key-phrase database

As stated, the preprocessing is done on a 'per-document' basis. The goal of the preprocessing step is to come up with a vector-based representation of each document from the original corpus. In this paper we evaluate two approaches for vector extraction: (1) plain *tf-idf *(term frequency - inverse document frequency) with low-weight cutoffs performed for each vector; (2) extracting possibly important frequent key-phrases and producing a vector representation for frequent keyphrases only. In either approach the focus is on the very simple assumption that the most relevant scientific articles for related concepts should contain similar vocabulary or keywords in the same way that related wikipedia entries share common categories, links, key-words, etc. The latter observation served as the basis for approaches presented in publications such as [21] and [22].

**Figure 2:**
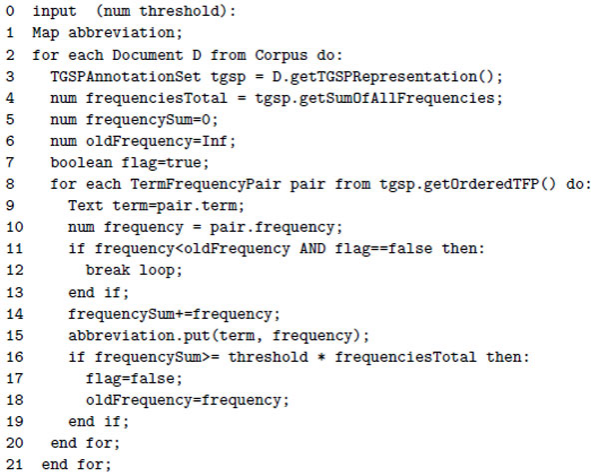
**T-GSP trimming**. Procedure for T-GSP based vector extraction.

For extracting candidate key-phrases from texts we have chosen a very simple T-GSP [23] approach, principally because it is efficient and easy to configure. T-GSP (Text General Sequential Pattern) is a text mining algorithm, which uses a single pass sliding window to extract the most frequent terms and their collocations from within a scope of a single document. T-GSP leverages shallow natural language processing techniques, i.e. part-of-speech tagging, to consider only those frequent candidates that fulfill one of the predefined grammar conditions. In the simplest case, one might consider a one element grammar that accepts only nouns ([*noun*], which can be fulfilled, for example, by a noun 'patient'). In a slightly more complex case, a grammar that accepts noun collocations may apply ([*noun, noun*], e.g. 'cancer patient'). Other grammars may accept [*noun, preposition, noun*] or [*noun, noun, noun*] collocations, etc. As a result, T-GSP generates a table of all candidate key-phrases sorted by their occurence frequency. At this point, in order to substantially reduce the computation space for further processing, we apply trimming, so that we include only a portion of the most frequent potential key-phrases in the document vector representation. The cutoff point here is defined as in line 16 of the pseudocode example presented in Figure 2. The size of the portion is determined in function of phrase frequency as related to the sum of all phrase frequencies from a given document. Key-phrases are stored and passed on in descending order by their frequencies. When the sum of the frequencies of the included phrases exceeds a threshold, the inclusion process continues until a less frequent key-phrase is encountered.

In the second part of the preprocessing procedure the key-phrases collected from each document are treated as a bag-of-words representation (denoted further as BOW), which means that each document generates a key-phrase based BOW. BOW of a document is a set of its unique terms with their frequency counts (term frequencies). The BOWs can be perceived as representations of the original documents. Each BOW is then represented as a vector in a Vector Space Model (VSM) of *tf-idf *weighted vectors. Weights for individual terms are calculated as in Formula 2. This means that BOWs are processed as if they were documents in a typical VSM. They are tokenized (after stopwords deletion) and in-document frequency is calculated for each meaningful token. The *tf *factor may be based either on the original document's frequency for the same token or on the frequency of the token inside the abbreviation. The *idf *factor is based on abbreviations vector space only. Each term token has a position assigned to it within the vector space. Vectors are stored as lists of position - value pairs (positive values only) in the vector DB (sparse vector representation), which is later used at runtime, together with the corpus index. For *tf *(*t, d*), where *t *denotes a term and *d *denotes a document, raw frequency is used. It can be obtained either from the BOW itself or from the general document. The inverse document frequency component is calculated as follows:

(1) $$idf\left(t,D\right)=\text{log}\frac{\left|D\right|}{|d\in D:t\in d|+1},$$

where *D *denotes the document corpus, *t *denotes the term and *d *a document. Those elements lead us to the formula for *tf-idf *:

(2) tfidf(t,d,D)=tf(t,d)×idf(t,D)

Alternatively (i.e. in the variant without T-GSP processing), vector representations of documents are produced directly through a traditional VSM approach with trimming applied individually for each vector at positions with lower statistical significance for the documents. In this approach *tf-idf *weights (as defined in Formula 2) are associated directly with the BOW representation of the document itself. As well as in the case of T-GSP generated BOWs, here too are tokenization, normalization and stopwords elimination applied to the process, through the mechanism provided by the Lucene StandardAnalyzer.

Note that cutoffs are defined differently for the two variants of the vector extraction method. In the T-GSP version the threshold is defined in the function of total frequencies of all possible key-phrase candidates, whereas the second variant simply includes a percentage of the most significant (in terms of higher weights) dimensions of each vector, leaving zeros in the positions not included.

### Runtime processing

As mentioned, runtime processing is executed for two terms/phrases being tested for relatedness. The procedure can be broken down into three steps: (1) establishing a set of the most relevant documents for each text, (2) aggregating vector representations of these documents into a vector for each input text, (3) calculating relatedness approximation based on a vector based metric. In this subsection we describe each of these steps in more detail. The first step, i.e. finding the most relevant documents for a given input, is resolved straightforwardly through standard mechanisms of the Lucene Index, through which the Document Corpus element from the overview provided in Figure 1 can be queried. The input texts are parsed as queries to the index, using the least restrictive approach for the Boolean retrieval (multi-term queries are represented as an alternative of terms). The results of the search are passed on as input for the second step. The number of the most relevant documents to be included in the output is a preset parameter, the implications of which are covered in more detail in the Results section of this paper. Aggregating a vector representation for a textual input is done by vector addition. At this point each of the input terms/phrases has an aggregated vector assigned to it to represent its approximated context. The issue of context approximation in the specific settings of scientific documents is discussed in more detail in the Results and discussion section of this paper. As for the actual calculation of the numerical approximation of the semantic relatedness, we decided to use cosine similarity.

### Experiments

The method has been evaluated in a series of experiments, the settings of which are described in this section. In each of the experiments presented, the method was used to produce a relatedness score for each input pair of textual labels. The number of pairs per experiment varies from 29 to 6091686. In order to evaluate the performance we have measured coverage. Coverage can indicate how well the method adapts to different scenarios without any specific recalibrations. Coverage can be defined for a space of single inputs:

(3) $$r_{s}\left(S\right)=1-\frac{\left|\left\{v\in V\left(S\right):|v|=0\right\}\right|}{\left|S\right|},$$

where *S *is a set of inputs, *r_s_*(*S*) denotes coverage over *S *and *V *(*S*) denotes a set of vectors generated for inputs *S*. This notion seems quite informative, as it corresponds to the percentage of inputs from a given set, that generate non-zero vectors. However, in the literature the most common notion is one of coverage based on input pairs found in a given dataset:

(4) $$r_{p}\left(S\right)=1-\frac{\left|\left\{\left(v_{i},v_{j}\right)\in V\left(S\right):|v_{i}|\;=\;0\;\text{V}|v_{j}|\;=\;0\right\}\right|}{\left|S\right|},$$

where *S *is a set of input pairs, *r_p_*(*S*) denotes coverage over *S *and *V *(*S*) denotes a set of vector pairs generated for inputs *S*.

In the case of direct evaluations, i.e. when comparing automatically obtained results with human judgement, the quality of the results has been reflected by measuring Spearman's rank correlation coefficients, with average values assigned for tied ranks, as defined in Formula 5:

(5) $$\rho \left(X,Y\right)=\frac{\sum_{i}\left(x_{i}-\bar{x}\right)\left(y_{i}-\bar{y}\right)}{\sqrt{{\left(x_{i}-\bar{x}\right)}^{2}{\left(y_{i}-\bar{y}\right)}^{2}}},$$

where *x_i _*and *y_i _*are ranks obtained for raw scores *X_i _*and *Y_i _*of variables *X *and *Y *.

Apart from the performance evaluation, the experiments were also used for testing different aspects of the method itself, i.e. its sensibility to parameters and corpus. As mentioned, the method depends on two input parameters, these being the candidate cutoff rate applied at the preprocessing stage and the number of relevant documents recovered for each input label. Additionally, every corpus-based method depends heavily on the choice of corpus, therefore the performance on two different corpora has also been reflected to some extent in the course of the experimental evaluation (full papers vs. abstracts only). The following subsection presents all the data used in the experiments, both as benchmarks and as document repositories.

#### Data

Our method has been evaluated directly with the following datasets: 566 label pairs rated by medical residents for semantic similarity (residents-s) and 587 pairs rated for relatedness (residents-r) [24], 101 label pairs ranked by medical coders (101c) for relatedness, set of 29 pairs rated by coders (29c) and physicians (29p) for relatedness [19]. All the datasets used as references [25] in the evaluation process are summarized in Table 1. Table 2 which has been compiled according to the study presented in [26] and results reported in [27], presents reported reference results for the benchmarks. To the best of our knowledge, this data is henceforth accurate. Nonetheless, and more importantly, it gives an idea as to how the state-of-the-art measures could perform across various reference standards.

**Table 1:** Presentation of the general characteristics of the datasets used in experiments; number of pairs and distinct items describe the size of the datasets; the reference column indicates whether a dataset is a reference benchmark; the focus of the dataset column contains the information on the type of relationships captured in the reference results.

	No of pairs	Distinct items	Reference	Focus of the dataset
residents-s	566	375	Yes	Similarity
residents-r	587	397	Yes	Relatedness
101c	101	191	Yes	Relatedness
29c	29	56	Yes	Relatedness
29p	29	56	Yes	Relatedness
orphanetDG	6091686	4937	No	-
orphanetDD	2941525	2426	No	-

**Table 2:** Presentation of the state-of-the art results reported in the literature for the reference benchmarks.

Benchmark	Reported correlation	Method class	Focus of the method	Citation
residents-s	0.46	Taxonomy-based	Similarity	[26]
residents-r	0.39	Taxonomy-based	Similarity	[26]
101c	0.46	Taxonomy-based	Similarity	[26]
29c	0.90	Taxonomy-based	Similarity	[27]
29p	0.84	Medical notes corpus; thesaurus preprocessing	Relatedness	[19]

It is worth noting, that methods and benchmarks can be focused on one of two distinct notions, i.e. semantic similarity and semantic relatedness. Semantic similarity tells us how two entities are similar to each other, while relatedness is a broader term, associated with any kind of semantic relationship between the entities of interest. E.g. *Clostridium perfringens *and *Gangrene *should display a low similarity, as one is a bacteria and the other is a health condition. At the same time those two entities should be semantically related, because *C. perfingens *is a microbe that can produce the condition, therefore this pair should have a high relatedness score. The difference between the notions is important for understanding particular results, nonetheless it would require very specific features in a benchmark to have an important effect in the course of the evaluation. Furthermore, the distinction between the two notions will often depend on a semantic perspective, and therefore might not be very clear to human annotators. In practice, the semantic relatedness approximation methods are often evaluated against similarity benchmarks and vice versa, which is partially reflected in Tables 1 and 2. We assume that taxonomy-based measures are all focused on similarity, as suggested in [27], as they are aimed at capturing taxonomical relationships only.

For each of the datasets mentioned in Table 2 the experiments were executed for both corpus versions (full vs abstracts-only) with different values of in-document cutoff rate (0.1 - 0.9, with a 0.1 step for the T-GSP variant; 0.05 - 0.4 with 0.05 step for the plain *tf-idf *variant). As for the document corpus, an open access subset of PubMedCentral [28], containing 453531 life sciences research articles, was chosen as the reference material. The corpus we have used was last updated in the spring of 2012, the currently available one is signifantly larger. For testing the robustness of the method with respect to the document corpus the method was set up with two versions of the corpus, full documents vs. abstracts-only. It is worth noting, that although we describe the corpus as 'moderately large' and 'fairly general', it actually contains almost half a million documents devoted to Life Sciences. Our phrasing is closely related to the size and scope of the domain, which contains fields as diverse as computational biology, chemistry and plastic surgery. Also, there is a massive number of Life Science publications, with more than 20 million article records in Medline [29]. Nonetheless, the size and nature of the corpus will depend on the application domain and specific use cases. Our assumption is, that for the presented method to work, it should be enough to consider a moderately large and fairly large sample of the publications from a given domain.

Additionally, the method has also been tested on a larger scale dataset, two experiments were performed: first, for pairwise comparisons of all possible of genes and disorders retrieved from OrphaNet dataset [30]; second, for disorder-disorder pairs, with data from the same dataset. Genes include all genes mentioned in the original dataset, while disorders are diseases believed to have a genetic background (they have at least one associated gene in the original dataset). The experiments involved 6091686 gene-disorder pairs and 2941525 disorder-disorder pairs, respectively (2511 distinct genes and 2426 distinct disorders represented by their names extracted from the original OrphanetData). Without additional human annotation, the quality of the method is not measurable in the OrphaNet experiment (mostly due to the 'openness' of the problem at hand), so more focus was placed on showing specific interesting results, both positive and negative in terms of the correctness of the approximation. In other words, it is not possible to evaluate the actual results obtained from the OrphaNet experiments, although some interesting examples from these experiments (that are potentially close to a real-world application of the method) are also presented in the next section.

The pairs extracted from the OrphaNet dataset and used in our experiments can be found in the results files, included as supplementary material, at [31].

## Results and discussion

The following section presents the results of the experimental evaluation of the method. The section is divided into three main parts: firstly, the results of direct evaluation are presented and discussed; then the OrphaNet experiment is examined. In the third part some general discussion points are presented, mainly those that are only loosely related to particular evaluation results. Table 3 presents statistics of vector-representation databases obtained for different settings.

**Table 3:** Statistics of different vector databases in the function of their setup parameters.

Variant	Threshold [%]	Vector size (mean)	Physical size	Composition
T-GSP	0.1	3.66	109 MB	Full articles
T-GSP	0.2	11.09	257 MB	Full articles
T-GSP	0.3	24.25	522 MB	Full articles
T-GSP	0.4	46.36	968 MB	Full articles
T-GSP	0.5	83.7	1.68 GB	Full articles
T-GSP	0.6	140.39	2.79 GB	Full articles
T-GSP	0.7	239.96	4.52 GB	Full articles
T-GSP	0.8	359.56	6.2 GB	Full articles
T-GSP	0.9	372.14	6.37 GB	Full articles
No T-GSP	0.05	38.31	852 MB	Full articles
No T-GSP	0.1	76.43	1.6 GB	Full articles
No T-GSP	0.15	114.09	2.36 GB	Full articles
No T-GSP	0.2	151.6	3.13 GB	Full articles
No T-GSP	0.25	189.29	3.89 GB	Full articles
No T-GSP	0.3	227.13	4.63 GB	Full articles
No T-GSP	0.35	264.92	5.31 GB	Full articles
No T-GSP	0.4	302.59	5,87 GB	Full articles

T-GSP	0.1	2.77	97 MB	Abstracts only
T-GSP	0.2	6.43	168 MB	Abstracts only
T-GSP	0.3	12.37	287 MB	Abstracts only
T-GSP	0.4	22.05	483 MB	Abstracts only
T-GSP	0.5	34.72	738 MB	Abstracts only
T-GSP	0.6	43.86	921 MB	Abstracts only
T-GSP	0.7	46.69	978 MB	Abstracts only
T-GSP	0.8	47	984 MB	Abstracts only
T-GSP	0.9	47.01	985 MB	Abstracts only
No T-GSP	0.05	4.9	147 MB	Abstracts only
No T-GSP	0.1	9.27	237 MB	Abstracts only
No T-GSP	0.15	13.69	327 MB	Abstracts only
No T-GSP	0.2	18	416 MB	Abstracts only
No T-GSP	0.25	22.37	506 MB	Abstracts only
No T-GSP	0.3	26.85	597 MB	Abstracts only
No T-GSP	0.35	31.28	688 MB	Abstracts only
No T-GSP	0.4	35.59	776 MB	Abstracts only

### Approximating human judgement

**Figure 3:**
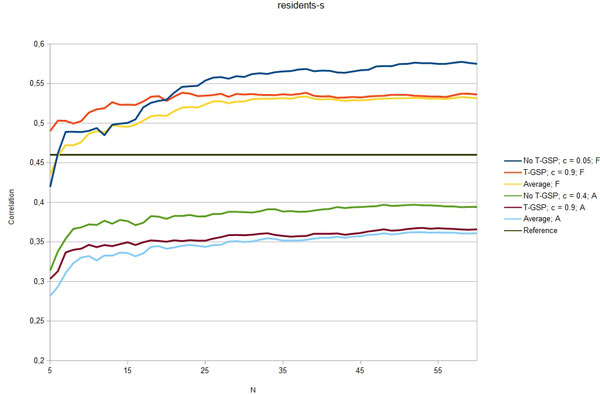
**Correlation with human judgement**. Selected results (correlation in the function of N documents aggregated per vector) for the residents-s dataset; legend indicates the vector extraction variant (T-GSP/No T-GSP), cutoff point *c *and corpus used (F - full corpus, A - abstracts-only); horizontal reference line correponds to the reference value presented in Table 2.

**Figure 4:**
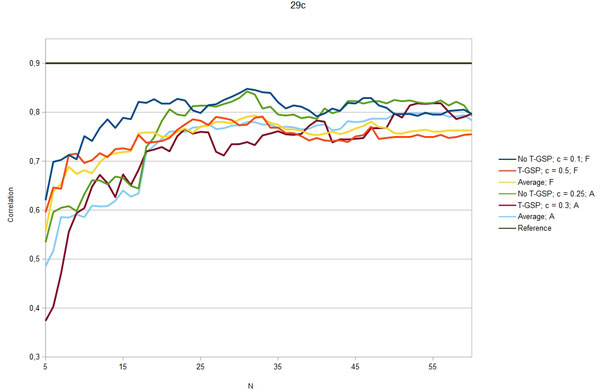
**Correlation with human judgement**. Selected results (correlation in the function of N documents aggregated per vector) for the residents-r dataset; legend indicates the vector extraction variant (T-GSP/No T-GSP), cutoff point *c *and corpus used (F - full corpus, A - abstracts-only); horizontal reference line correponds to the reference value presented in Table 2.

**Figure 5:**
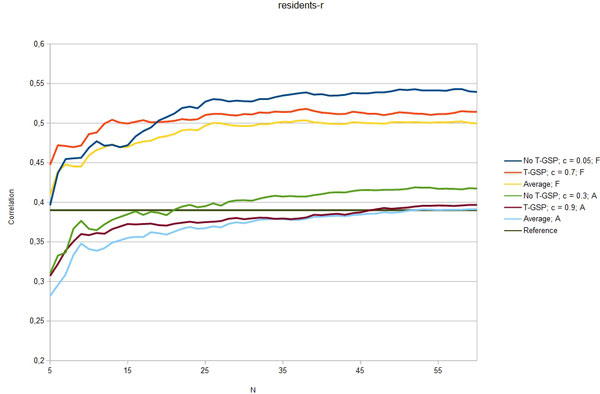
**Correlation with human judgement**. Selected results (correlation in the function of N documents aggregated per vector) for the29c dataset; legend indicates the vector extraction variant (T-GSP/No T-GSP), cutoff point *c *and corpus used (F - full corpus, A - abstracts-only); horizontal reference line correponds to the reference value presented in Table 2.

**Figure 6:**
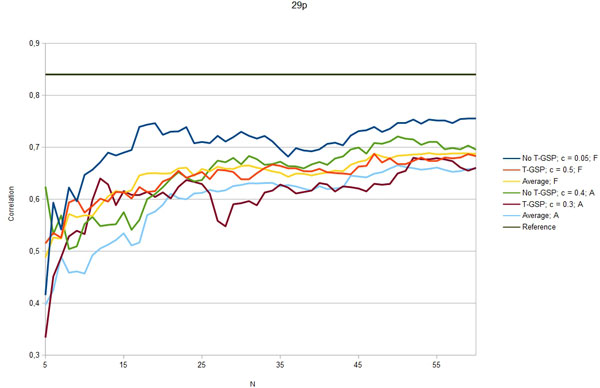
**Correlation with human judgement**. Selected results (correlation in the function of N documents aggregated per vector) for the 29p dataset; legend indicates the vector extraction variant (T-GSP/No T-GSP), cutoff point *c *and corpus used (F - full corpus, A - abstracts-only); horizontal reference line correponds to the reference value presented in Table 2.

**Figure 7:**
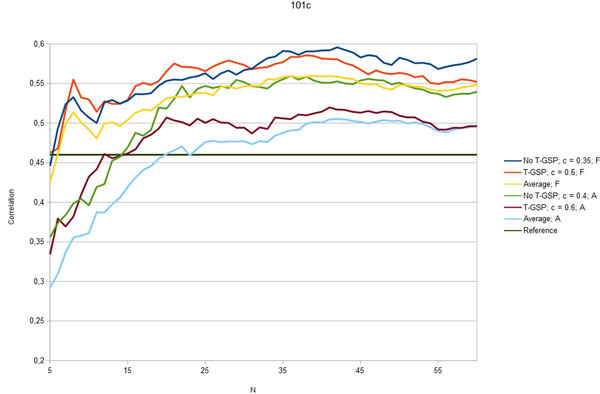
**Correlation with human judgement**. Selected results (correlation in the function of N documents aggregated per vector) for the 101c dataset; legend indicates the vector extraction variant (T-GSP/No T-GSP), cutoff point *c *and corpus used (F - full corpus, A - abstracts-only); horizontal reference line correponds to the reference value presented in Table 2.

Figures 3 - 7 show the Pearson correlation coefficient of the results vector with average human answers in the function of number of relevant documents aggregated as vectors for each input. Each figure corresponds to one of the benchmarks and each includes six plots: best T-GSP, best without T-GSP and average results; all three for each of the corpus versions (full papers and abstracts only). Average and best values for different vector database settings are presented in Table 4.

**Table 4:** Correlation with human judgement obtained for various combinations of vector extraction variant, trimming threshold and corpus.

Parameters	residents-s		residents-r		29p		29c		101c	
Extr.; c; Corpus	Max (N)	Avg	Max (N)	Avg	Max (N)	Avg	Max (N)	Avg	Max (N)	Avg
T-GSP; c = 0.9; F	0.54 (38)	0.53	0.51 (27)	0.5	0.68 (57)	0.63	0.79 (31)	0.74	0.58 (39)	0.55
T-GSP; c = 0.8; F	0.54 (38)	0.53	0.51 (26)	0.5	0.68 (57)	0.62	0.77 (47)	0.73	0.58 (39)	0.55
T-GSP; c = 0.7; F	0.54 (58)	0.52	0.52 (38)	0.51	0.68 (56)	0.63	0.79 (32)	0.74	0.58 (40)	0.55
T-GSP; c = 0.6; F	0.52 (58)	0.51	0.5 (37)	0.49	0.66 (60)	0.63	0.78 (24)	0.74	0.59 (38)	0.56
T-GSP; c = 0.5; F	0.53 (38)	0.52	0.5 (37)	0.49	0.69 (47)	0.64	0.79 (33)	0.74	0.55 (29)	0.53
T-GSP; c = 0.4; F	0.52 (38)	0.51	0.5 (26)	0.49	0.65 (57)	0.6	0.75 (25)	0.7	0.56 (27)	0.52
T-GSP; c = 0.3; F	0.49 (41)	0.47	0.47 (55)	0.45	0.65 (57)	0.6	0.75 (29)	0.7	0.53 (31)	0.5
T-GSP; c = 0.2; F	0.47 (52)	0.44	0.44 (58)	0.4	0.64 (17)	0.58	0.77 (32)	0.71	0.51 (23)	0.48
T-GSP; c = 0.1; F	0.42 (37)	0.38	0.38 (57)	0.34	0.61 (25)	0.47	0.75 (26)	0.6	0.42 (34)	0.36
T-GSP; -; F	0.54	0.49	0.52	0.46	0.69	0.6	0.79	0.71	0.59	0.51
No T-GSP; c = 0.4; F	0.56 (34)	0.55	0.52 (26)	0.51	0.73 (47)	0.69	0.82 (32)	0.77	0.6 (42)	0.56
No T-GSP; c = 0.3; F	0.56 (38)	0.55	0.53 (38)	0.51	0.73 (47)	0.69	0.82 (32)	0.78	0.59 (42)	0.56
No T-GSP; c = 0.35; F	0.56 (34)	0.55	0.53 (26)	0.51	0.73 (47)	0.69	0.81 (31)	0.78	0.6 (42)	0.56
No T-GSP; c = 0.2; F	0.57 (58)	0.55	0.53 (38)	0.52	0.74 (60)	0.7	0.84 (32)	0.79	0.59 (39)	0.56
No T-GSP; c = 0.25; F	0.56 (58)	0.55	0.53 (38)	0.52	0.74 (54)	0.69	0.82 (33)	0.78	0.59 (42)	0.56
No T-GSP; c = 0.1; F	0.57 (58)	0.55	0.54 (38)	0.52	0.75 (59)	0.71	0.85 (31)	0.8	0.59 (36)	0.55
No T-GSP; c = 0.15; F	0.57 (58)	0.55	0.53 (38)	0.52	0.75 (54)	0.7	0.84 (32)	0.79	0.59 (43)	0.56
No T-GSP; c = 0.05; F	0.58 (58)	0.55	0.54 (58)	0.52	0.76 (59)	0.7	0.85 (33)	0.8	0.59 (39)	0.55
No T-GSP; -; F	0.58	0.55	0.54	0.52	0.76	0.7	0.85	0.79	0.6	0.56
-; -; F	0.58	0.52	0.54	0.49	0.76	0.64	0.85	0.75	0.6	0.53

T-GSP; c = 0.9; A	0.37 (53)	0.35	0.4 (59)	0.38	0.66 (26)	0.63	0.82 (55)	0.77	0.51 (42)	0.48
T-GSP; c = 0.8; A	0.37 (53)	0.35	0.4 (59)	0.38	0.66 (26)	0.63	0.82 (55)	0.77	0.51 (42)	0.48
T-GSP; c = 0.7; A	0.37 (53)	0.35	0.4 (59)	0.38	0.66 (24)	0.63	0.82 (53)	0.78	0.51 (42)	0.48
T-GSP; c = 0.6; A	0.36 (55)	0.35	0.39 (59)	0.37	0.66 (25)	0.63	0.82 (24)	0.77	0.52 (41)	0.49
T-GSP; c = 0.5; A	0.35 (56)	0.34	0.39 (59)	0.37	0.66 (24)	0.62	0.8 (24)	0.75	0.51 (41)	0.48
T-GSP; c = 0.4; A	0.35 (52)	0.34	0.38 (56)	0.36	0.67 (52)	0.63	0.8 (57)	0.75	0.46 (48)	0.43
T-GSP; c = 0.3; A	0.32 (57)	0.31	0.37 (58)	0.35	0.68 (52)	0.61	0.82 (56)	0.72	0.45 (51)	0.42
T-GSP; c = 0.2; A	0.32 (57)	0.31	0.35 (57)	0.33	0.6 (50)	0.46	0.74 (26)	0.62	0.46 (41)	0.39
T-GSP; c = 0.1; A	0.28 (57)	0.25	0.28 (60)	0.24	0.62 (49)	0.47	0.7 (48)	0.57	0.38 (52)	0.26
T-GSP; -; A	0.37	0.33	0.4	0.35	0.68	0.59	0.82	0.72	0.52	0.43
No T-GSP; c = 0.4; A	0.4 (48)	0.38	0.42 (52)	0.4	0.72 (50)	0.65	0.83 (50)	0.78	0.56 (36)	0.52
No T-GSP; c = 0.3; A	0.4 (52)	0.38	0.42 (52)	0.4	0.71 (50)	0.64	0.83 (56)	0.77	0.55 (36)	0.51
No T-GSP; c = 0.35; A	0.4 (52)	0.38	0.42 (53)	0.4	0.72 (50)	0.64	0.83 (50)	0.77	0.55 (36)	0.51
No T-GSP; c = 0.2; A	0.39 (53)	0.37	0.42 (53)	0.39	0.69 (50)	0.6	0.84 (32)	0.75	0.56 (42)	0.51
No T-GSP; c = 0.25; A	0.39 (52)	0.37	0.42 (53)	0.39	0.71 (49)	0.64	0.84 (31)	0.77	0.55 (42)	0.51
No T-GSP; c = 0.1; A	0.38 (57)	0.36	0.41 (60)	0.38	0.68 (60)	0.57	0.82 (58)	0.71	0.55 (53)	0.49
No T-GSP; c = 0.15; A	0.38 (53)	0.36	0.41 (53)	0.38	0.69 (60)	0.59	0.83 (32)	0.74	0.55 (43)	0.49
No T-GSP; c = 0.05; A	0.37 (34)	0.34	0.41 (34)	0.37	0.69 (60)	0.5	0.83 (58)	0.66	0.52 (59)	0.43
No T-GSP; -; A	0.4	0.37	0.42	0.39	0.72	0.6	0.84	0.74	0.56	0.49
-; -; A	0.4	0.35	0.42	0.37	0.72	0.6	0.84	0.73	0.56	0.46

The table presents both average and best results obtained for each combination of parameters. For best results, information about N value (number of aggreagted documents) is also included. Parameter c is the trimming threshold, while F denotes 'Full corpus' and A stands for 'Abstracts only'. '-' denotes an aggregation (max/avg) over all parameter values.

**Figure 8:**
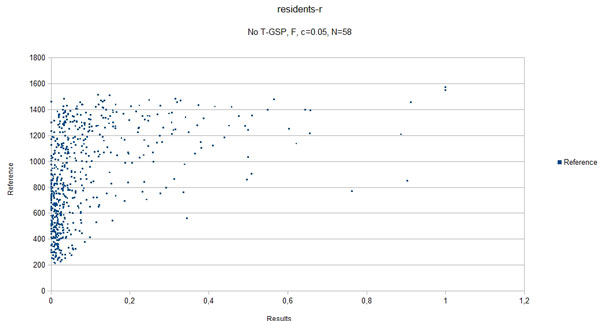
**Scatter plot, full corpus**. A scatter plot of the best case scenario, full corpus (correlation coefficient *ρ *= 0.55). The plot shows the results obtained for the residents-r datasets in the funtion of reference values.

**Figure 9:**
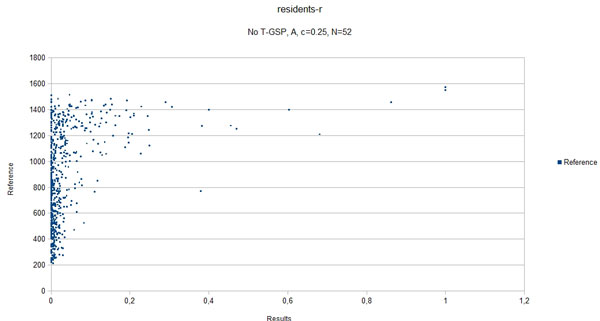
**Scatter plot, abstracts only**. A scatter plot of the best case scenario,abstracts only (correlation coefficient *ρ *= 0.42). The plot shows the results obtained for the residents-r datasets in the funtion of reference values.

In the presented method, coverage depends on the corpus only, therefore its value is the same across the different databases for each of the respective datasets. The coverage values are summarized in Table 5. Figure 8 shows the relationship between the results of our method and average human answers in the best-case scenario for the residents-r dataset. Figure 9 shows the same relationship obtained for the best-case settings for the compact corpus (abstracts only).

**Table 5:** Coverage (recall) recorded for different reference datasets.

Dataset:	residents-s		residents-r		29p		29c		101c	
Corpus:	F	A	F	A	F	A	F	A	F	A
r_p_:	0.95	0.77	0.94	0.75	1.0	1.0	1.0	1.0	0.97	0.92
r_s_:	0.97	0.85	0.96	0.83	1.0	1.0	1.0	1.0	0.98	0.96

The results obtained clearly show that, given the state-of-the-art results cited in Table 2 the proposed method is capable of performing on a level comparable to other methods, outperforming them in 3 out of 5 of the reference datasets with some of the possible configurations (see Figures 3 - Figure 7), thus adding a new perspective to the evaluation presented in [26]. More importantly, the 'good' trimming threshold and document number configurations, given a corpus, seem to work for different reference datasets. There is a certain drop in quality of results with the abstracts-only corpus, more significant for the residents datasets. It can be partially explained by data insufficiency, as for the datasets in question it is also accompanied by a plunge in coverage reflected in Table 5. Our choice was to model the inability to retrieve a vector representation of the input with a zero relatedness score. The interpretation of this choice is, that when a concept does not appear in the corpus, there is a good chance that it is unrelated to the concepts that do appear in the corpus. Although not perfect, some assumptions have to be made in this 'null interpretation' issue. The one presented here, results in the scores of the method becoming even more bottom-loaded with the decrease of coverage, which is reflected in Figures 8 and Figure 9. Nonetheless, the results for the abstracts-only corpus can still be considered as fairly good, especially given the relative sizes of the respective vector DBs. One way of solving the coverage issue could be to use the compact corpus for the vector DB construction and full corpus for retrieval of the most relevant documents, when both versions of the corpus are available, but time/resource constraints are important.

When it comes to the T-GSP vector extraction vs plain *tf-idf *vector extraction, one can see that plain *tf-idf *seems to work slightly better, at least without additional optimization of the T-GSP parameters, which is beyond the scope of the work presented here. Nonetheless T-GSP extraction results in a method that still provides results comparable with those of the state-of-the-art methods, therefore it performs reasonably well in terms of noise reduction. Its interesting property is that the documents can be processed independently up to the point at which the trimming is performed (trimming is applied locally at document level) and it does not use the statistics of the entire corpus until this moment. As a result, the *idf *part of *tf-idf *scheme is computed after the trimming has been done. All this makes the T-GSP variant potentially more appealing in terms of parallelism and memory efficiency.

### Data analysis

Potential data analysis application of the method is presented through example experiments with the OrphaNet dataset, which involves relatedness calculations for pairs of terms/phrases related to orphaned and rare diseases. Two experiments have been carried out, for disorder - gene (orphanetDG) and disorder - disorder (orphanetDD) relatedness. The same setup of the method has been used for both experiments: full article corpus, aggregation of 20 documents per input, preprocessing without T-GSP, with trimming threshold set at 0.05. The method performed well regarding recall, as it managed to retrieve representation vectors for 99.86% and 99.71% of the inputs involved in the respective experiments. Tables 6 and 7 present some of the results obtained in the course of the experiments. In each of the tables rows 1-3 present potentially relevant results, while rows 4-5 illustrate a visible flaw of the approach in the OrphaNet experiments. Full results of the OrphaNet experiments can be accessed at [31].

**Table 6:** Selection of interesting results obtained from the orphanetDG experiment.

	*T*_1_	*T*_2_	*ρ*(*T*_1 _*, T*_2 _)
1	Bardet-Biedl syndrome	Bardet-Biedl syndrome 1	1.0
2	Birt-Hogg-Dube syndrome	Folliculin	0.95
3	Facioscapulohumeral dystrophy	FSHD region gene 1	0.95
4	Haim-Munk syndrome	Alstrom syndrome 1	0.38
5	Autosomal recessive Stickler syndrome	Usher syndrome 1C (autosomal recessive, severe)	0.34

In the positive examples, the approach works as expected. The negative examples illustrate one of the differences between knowledge-based and distributional methods for approximating relatedness between concepts or their lexical representations. The method might display undesired behavior when input collocation of words share common tokens. Obviously, this might occur in a 'true positive' situation (Table 6 row 1), but sometimes inputs will produce a high score because they are tied by a common token of little significance. An example is presented in Table 7 row 4. The inputs share the word 'syndrome', while the other terms of both inputs fail to influence the retrieval of the most relevant documents, as they are most probably not present in the corpus. This problem can be dealt with by adding more elements to the method. Solutions could include adding more restrictive boolean filtering at the point of document retrieval or using query-document weights to reduce the impact of low importance documents at the point of vector aggregation. Both solutions were tried in the course of the direct evaluation, but in the case of the reference datasets they only resulted in a slight decrease in the quality of the results.

**Table 7:** Selection of interesting results obtained from the orphanetDD experiment.

	*T*_1_	*T*_2_	*ρ*(*T*_1_*, T*_2_)
1	Pentosuria	Argininemia	0.97
2	Leukonychia totalis	Blepharophimosis - epicanthus inversus - ptosis	0.95
3	Ellis Van Creveld syndrome	Postaxial acrofacial dysostosis	0.79
4	Clouston syndrome	Crisponi syndrome	0.98
5	Autosomal dominant vitreoretinochoroidopathy	Autosomal dominant macrothrombocytopenia	0.98

This issue, although it seems to be related to data sparseness, also points to a deficiency, characteristic of all lexical distributional proxies for semantic relatedness. One may notice, that the methods in this class are not able to cope with synonymy and polysemy, as the representations of concepts are assigned in the funtion of their lexicalizations, i.e. the input texts. Although this is true, it is also worth noting that neither of the KB-based methods resolve this problem without additional information, i.e. it is not possible to correctly disambiguate an ambiguous textual input to a specific taxonomy node without contextualizing the input (e.g. by supplying a unique identifier). The architecture of our method enables a seamless incorporation of additional information available at runtime, as it can be done through reformulating the queries in STEP 1 of the method (e.g. by using non-ambiguous synonyms in the case they are available). This strategy seems reasonable in knowledge poor settings as in many cases it could work with partial or incomplete knowledge. We assume that evaluating this approach is an interesting line for future research. On the other hand, when no additional information is available, polysemy can be inferred through context mining, as suggested, for example, in [32] or more recently in [33].

Having said which, the output of the OrphaNet experiments does present value for the data analysis. Depending on the actual needs of the final user, different heuristic strategies of results filtering can be employed. For example, rows 2-3 of Table 6 and rows 1-3 of Table 7 were extracted with a script that returns high scoring pairs with no common tokens. If the actual task was to create relatedness clusters for an application such as query expansion, there is a good chance that replacing the scores for the entities that share common tokens with a *null */missing value would still produce good clustering results.

### General remarks

The method presented here relies heavily on the idea that related concepts/phrases share common contexts. Moreover, it was assumed that it would be enough to roughly approximate the contexts, by representing them with selection of the most important ones only. Although this approach does display flaws, characteristic to distributional methods (as shown by the OrphaNet examples), some of them can be dealt with (or controlled to some extent) by fine tuning the method for given usage scenarios. Tuning a general method in many cases is still a lot more feasible than creating a comprehensive, domain-spanning knowledge base with an extensive lexical layer, which in many cases is either impossible or requires a substantial effort on the part of the community. Thus, in knowledge-poor environments the corpus-based methods can produce reasonably good results, given an adequate document collection.

The experimental evaluation has shown that using research documents/abstracts for context approximation can lead to good evaluation results. One hypothesis, potentially to be pursued in future work, is that the good quality of the results is related to the properties of research articles and research corpora. A research article, although technically is processed as free-text, does actually have a fairly rigid structure and use of words is much more restrictive than in general domain texts. It is also worth pointing out that the open subset of PMC is an ever growing full article corpus, whose potential often seems to be overlooked.

While it is true that distributional measures are corpus dependent, this does not have to be seen as a flaw. This characteristic means that distributional measures are more likely to evolve with a domain of knowledge, as corpora follow the developement of a domain much more naturally than thesaurus-like resources. In fairly static domains this might be less important, but in the dynamic fields, such as genomics or metabolomics, where the panorama shifts quickly, the distributional methods will not only be more usable, but will also cater to different needs. For example, a measure coupled with an evolving corpus can be used to monitor changes in concept/word relatedness over time, something which is complicated to model with other approaches.

Overall, our impression is that, even in the static cases, the corpus dependent methods are easier to use and adapt, unless there is an available knowledge base suitable for a specific application scenario. At the same time, the available Life Sciences databases are often insufficient and it is unrealistic to assume that there will be KB representations adequate for each specific case that involves semantic relatedness, whereas the research papers are published on an extremely wide variety of problems and subproblems. Therefore, scientific texts contain knowledge, which is less dependent on a specific perspective of a given KB, whereas the structured sources, such as ontologies, often represent only a very specific vision of a domain, e.g. modeled through the taxonomic relationships. Because of that, the distributional methods will be more likely to capture a wide variety of semantic relationships between the objects, regardless of the structured description of the domain. Additionally, scientific texts in an up-to-date corpus also contain knowledge on newly minted entities, as opposed to the formalised domain descriptions, which tend to follow with a delay. Whether or not free text is the best way of representing the knowledge acquired through research, constitutes a separate discussion point, raised for example in [34]. Nonetheless, this is the current state of the academic publishing and it might not change in the immediate future. Therefore, it is essential to provide the basic enabling techniques that work in knowledge-poor settings.

## Conclusions

In this paper we have presented a corpus-based methodology for calculating an approximation for lexical semantic relatedness for use in the life sciences domain in knowledge poor settings. Its quality and properties have been demonstrated through direct evaluation, i.e. tests with reference datasets that contain term/phrase pairs scored by human annotators. The method outperformed the state-of-the-art solutions in 3 out of 5 reference datasets. Additionally, it has been used in an open experiment with label pairs extracted from the OrphaNet database. The results of the experiments have been included as Additional file 1 with the larger files, related to the OrphaNet experiments, made available at [31].

## List of abbreviations used

BOW - bag of words

KB - knowledge base

NLP - natural language processing

PMC - PubMed Central

T-GSP - Text General Sequential Pattern

*tf-idf *- term frequency - inverse document frequency

VSM - vector space model

## Competing interests

The authors declare that they have no competing interests.

## Authors' contributions

Both authors contributed to design of the method and expriments. MR was responsible for the implementation, performing the experiments and writing of the manuscript. Both authors read and approved the final manuscript.

## Supplementary Material

Additional file 1 **Results obtained for reference datasets**. The file contains the raw results obtained for the 809 unique label pairs that appear within all 4 reference datasets. The results were obtained for the method based on an entire corpus (full articles) with the trimming threshold of 0.05, with 58 documents aggregated per input. Please note that the OrphaNet result files, which are too large for being included in the publishing process, can be accessed at [31]. Click here for file

## Declarations

Publication costs for this article were funded by grants TIN2011-25840 (Ministerio de Ciencia e Innovación) and P11-TIC-7529 and P12-TIC-1519 (Plan Andaluz de Investigación, Desarrollo e Innovación).

This article has been published as part of *BMC Bioinformatics *Volume 15 Supplement 14, 2014: Semantic, social, mobile applications for bioinformatics and biomedical laboratories: selected works from the 13th International Workshop on Network Tools and Applications in Biology (NETTAB 2013). The full contents of the supplement are available online at http://www.biomedcentral.com/bmcbioinformatics/supplements/15/S14.
